# Pankey Mann Schuyler Philosophy-Based Prosthetic Rehabilitation for an Amelogenesis Imperfecta Patient: A Case Report

**DOI:** 10.7759/cureus.48395

**Published:** 2023-11-06

**Authors:** Arunoday Kumar, Babina Chirom, Ishani Ningthoujam, Rajesh S Nongthombam, Naorem S Kumar Singh

**Affiliations:** 1 Department of Prosthodontics and Crown & Bridge, Dental College, Regional Institute of Medical Sciences, Imphal, IND; 2 Department of Prosthodontics and Crown & Bridge, Prosthodontics Consultancy, Imphal, IND; 3 Department of Prosthodontics and Crown & Bridge, Dental College, Jawaharlal Nehru Institute of Medical Sciences, Imphal, IND

**Keywords:** porcelain fused to metal (pfm), all ceramic crowns, pankey mann schuyler (pms) philosophy, prosthetic rehabilitation, amelogenesis imperfecta (agi)

## Abstract

This clinical case report presents the prosthetic rehabilitation of a 23-year-old male patient with generalized discolored and worn-out teeth, which were of aesthetic and functional concern. In collaboration with the Department of Oral Medicine and Radiology and Oral Pathology, this clinical condition was diagnosed as amelogenesis imperfecta (AGI). AGI is a genetic odontological disorder that is an epithelial derivative of the developed tooth bud with enamel malformation. AGI typically affects both deciduous and permanent teeth. Patients generally have aesthetic complaints and compromised chewing efficiency with loss of vertical dimension. Prosthetically rehabilitating an AGI patient is a multidisciplinary approach to regain aesthetics, phonetics, and mastication.

This article describes the full mouth rehabilitation, following the Pankey Mann Schuyler philosophy, of the patient with AGI involving all teeth. Full mouth rehabilitation was planned to restore aesthetics, phonetics, and mastication in four phases. First was prosthetic rehabilitation of the mandibular anterior teeth, followed by the maxillary anterior, mandibular posterior, and, finally, maxillary posterior teeth.

## Introduction

Amelogenesis imperfecta (AGI) is a group of inherited disorders of tooth development characterized by disturbance in the ectoderm, where the malformation of enamel occurs [1,2]. In these cases, the teeth become weaker than normal and more susceptible to rapid wear, tear, and loss. In AGI, both deciduous and permanent detention are affected. AGI is classified into three broad groups, namely, hypoplastic type I, hypocalcification type II, and hypomaturation type III. These types of AGI occur at different stages of tooth development [3,4]. The hypoplastic type has a defective matrix; the hypocalcification type occurs after the formation of the matrix, where defective mineralization occurs. The last type, hypomaturation, is a condition where there is an immature formation of enamel crystallites [5].

Various problems are associated with AGI, depending on its type. Common problems include hypersensitivity, discoloration of teeth, and loss of vertical dimension (VD). Several approaches can be used to restore the aesthetics and normal functionality of teeth in AGI patients [6]. In this case, the patient was reported to have hypoplastic type II AGI, as confirmed by clinical, radiological, and histopathological examination. He was referred by the Department of Oral Medicine and Radiology and was treated with full mouth rehabilitation following the Pankey Mann Schuyler (PMS) approach.

Various occlusal schemes are available for full mouth rehabilitation. PMS, Hobo’s twin table, and Hobo’s twin stage are a few of the available schemes. Of these, PMS and Hobo’s twin stages are widely used [7]. The PMS technique is preferred over Hobo’s because it is a well-organized, logical procedure that progresses smoothly with less strain on the operator, patient, and technician. It divides the procedure into a series of separate appointments: first, permanent prosthetic rehabilitation of the anterior teeth, followed by posterior tooth rehabilitation. Laboratory procedures are short and simpler, and there is no need for time-consuming techniques and complicated instruments as required in other full mouth rehabilitation schemes. Moreover, the PMS philosophy is a flexible concept as the incisal guidance is not governed by condylar guidance rather posterior occlusal plane is based on the anterior guidance for the best esthetics, phonetics, function, and comfort of the patient [7].

In Hobo’s twin table technique, first, the posterior occlusal morphology is reproduced based on the condylar guidance recorded from the patient, followed by the reproduction of anterior morphology based on anterior guidance. The demerit of this technique is that the patient is not comfortable as the steep posterior cuspal angle makes the incisal guide table be set at a steep angle too, which can affect the morphology of anterior teeth. Moreover, Hobo’s twin table is technique sensitive [7]. However, in Hobo’s twin-stage technique, the occlusal morphology is reproduced based on standard cusp angle, and, hence, the record of condylar guidance from the patient is not required. Anterior morphology of the crown is made depending on acceptable incisal guidance, which would fit best as per esthetics phonetics, function, and comfort of the patient. The limitation of Hobo’s twin-stage technique is that it cannot be used for maloccluded teeth [7].

The objective of this case was full mouth rehabilitation of the patient with all-ceramic and porcelain-fused-to-metal (PFM) crowns and bridges, following the PMS philosophy [8]. The malformed teeth of the patient were restored to their natural form, function, and appearance while maintaining physiological integrity in a harmonious relationship with the adjacent hard and soft tissues of the stomatognathic system. This improved the patient’s oral health, quality of life, and social well-being.

## Case presentation

**Figure 1:**
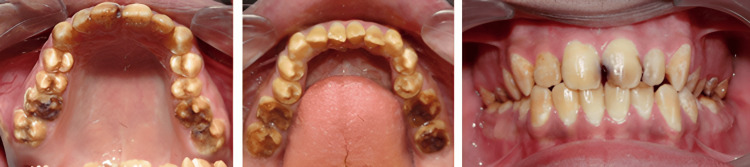
Preoperative intraoral view.

This clinical case report presents a 23-year-old male patient with the chief complaint of severely worn-out and discolored teeth with compromised aesthetics and chewing efficiency (Figure 1).

**Figure 2:**
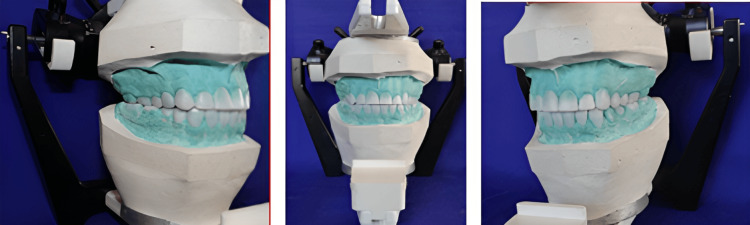
Diagnostic wax mockup at an increased vertical dimension of 2 mm following Broadrick’s occlusal flag analysis.

He did not provide any significant systemic medical history. On extraoral examination, he did not have any facial, muscular, or bony deformities. On clinical examination, the temporomandibular joint (TMJ) had well-coordinated and symmetrical movement without pain or crepitus. His physical growth in terms of height, width, stature, and appearance was within the normal range for his age group. He did not provide any relevant known history of allergy to any medicine or any adverse drug reaction. The intraoral examination showed generalized discolored (yellowish-brown) teeth with reduced VD (freeway space of 6 mm) due to wearing out of the occlusal surface. The patient also presented with dental caries with respect to 16 and short clinical crowns with respect to 36, 37, 46, and 47 as the teeth were worn out. Full mouth rehabilitation following the PMS philosophy with a multidisciplinary approach was planned for the patient. Oral prophylaxis, restorative therapy of 16 teeth, and crown lengthening of 36, 37, 46, and 47 preceded the prosthetic rehabilitation. The VD increased by 2 mm from the existing collapsed bite to regain the natural freeway space of 4 mm. The diagnostic mounting was done on a semi-adjustable articulator with the help of Hanau Springbow earpiece face-bow, taking a posterior interocclusal check record (thickness of 2 mm) and an anterior jig as a guide for mounting at an increased VD of 2 mm. A diagnostic wax mockup was created to re-establish the anterior and posterior occlusal planes. Anterior waxing was based on the aesthetics and phonetics of the patient, which were in harmony with the anterior guidance and condylar guidance. A mandibular posterior wax mockup was created using Broadrick’s occlusal flag analysis [9], which was also compatible with the anterior guidance and condylar guidance (Figure 2).

Putty index created from the wax mockup of the diagnostic cast

In this case, we used the PMS philosophy to perform the necessary treatment for full mouth rehabilitation. The treatment is divided into four steps. The first step is proper examination, diagnosis, and treatment planning for a better prognosis. The second is the determination of anterior incisal guidance for the best possible aesthetics, phonetics, function, and comfort of the patient. The third is the selection of an acceptable occlusal plane (by Broadrick’s flag analysis) and restoration of the lower posterior occlusion in harmony with the anterior guidance, without hampering or interrupting the condylar guidance [7].

To determine the mandibular occlusal plane, Broadrick’s flag analysis was performed using a caliper set at a radius of 4 inches from the needle point to the pencil point. The needle point of the caliper was set against the mandibular canine tip, and a line was drawn on the flag (anterior survey line). Then, the caliper point was held against the condylar ball of the articulator, and another arc was drawn on the flag (condylar survey line). From the intersecting point, a line was drawn from the molar to the canine. Using this method, we found the acceptable occlusal plane for the mandibular posterior teeth, which was in harmony with the curve of Spee. The fourth step in treatment is the restoration of the upper posterior teeth so that they have good intercuspation with the lower posterior teeth without any interferences or deflective contacts, which, in turn, is in harmony with the anterior guidance and condylar guidance.

**Figure 3:**
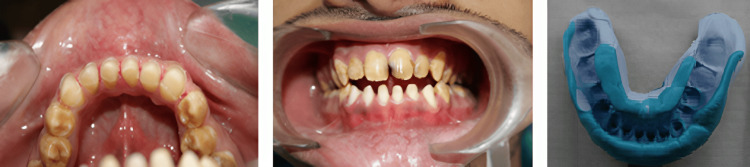
Preparation of the mandibular anterior teeth and final impression.

First, preparation of the mandibular anterior teeth was performed at the first appointment, as recommended for all-ceramic crowns (IPS Empress) for anterior teeth (Figure 3).

Minimal incisal tooth preparation was done as the teeth were already attrited due to the existing condition of AGI. The final impressions of the prepared maxillary and mandibular teeth were made with putty and light-body consistency addition silicone elastomeric impression material, following the two-step/double mix putty wash impression technique. The final impression was poured with a die stone to get the master cast.

**Figure 4:**
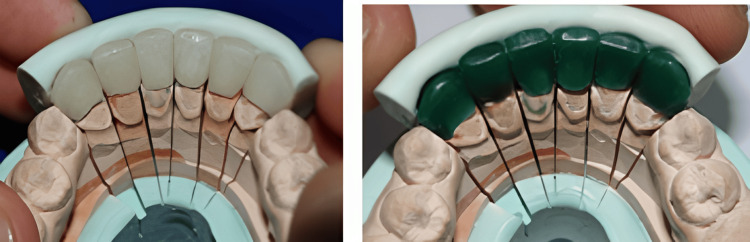
Putty index as a guide to fabricate anterior all-ceramic crowns.

Provisional crowns for the prepared anterior teeth were fabricated using Protem Plus™ as a temporary restorative material with the help of the putty index (made from the diagnostic wax mockup cast), following the direct fabrication technique. Provisional crowns were temporarily cemented with Temp-Bond™ until permanent all-ceramic crowns were fabricated in the laboratory using the same putty index as a guide (Figure 4).

**Figure 5:**
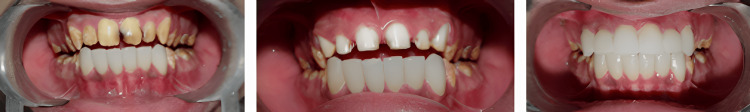
Permanent cementation of mandibular anterior all-ceramic crowns, followed by maxillary anterior tooth preparation and permanent cementation based on aesthetics and phonetics.

All-ceramic crowns, once fabricated, were cemented with permanent resin cement (3M RelyX™) after etching the prepared anterior teeth with phosphoric acid and etching the tissue surface of the all-ceramic crowns with hydrofluoric acid as recommended. After the cementation of all-ceramic crowns to the mandibular anterior teeth, maxillary anterior teeth were prepared in the second appointment. Temporary crowns were given, followed by permanent cementation of the maxillary all-ceramic crowns onto the prepared teeth. After cementation of both mandibular and maxillary anterior crowns, the patient was assessed for aesthetics, phonetics, and functional harmony (Figure 5). The anterior prosthesis was in harmony with the patient’s incisal guidance and condylar guidance.

**Figure 6:**
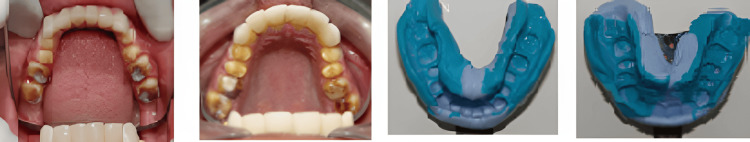
Mandibular and maxillary tooth preparation and final impression.

Following anterior cementation of the permanent prosthesis, the mandibular and maxillary posterior teeth were prepared in the third appointment, a final impression was made, and a master cast was poured (Figure 6).

**Figure 7:**
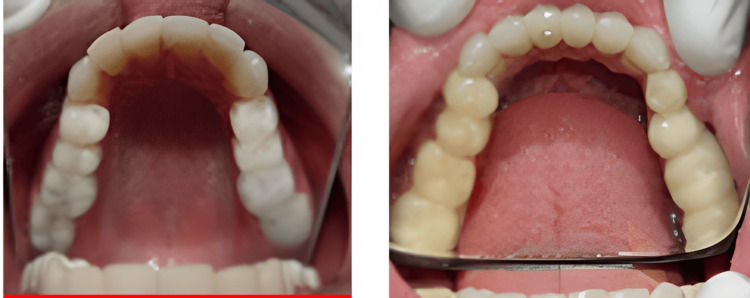
Temporization of the prepared posterior teeth.

Temporization of the prepared posterior teeth (using Protem as a temporary crown material) was performed at an increased VD of 2 mm (Figure 7).

Temporization was done by using a putty index (made from the diagnostic mockup cast) as a guide to fabricate temporary crowns and was kept for four weeks to look for any TMJ discomfort or masticatory muscle spasm (due to the 2 mm increase in VD). The patient did not report any TMJ or muscle spasms even after four weeks of temporary or provisional restorations. The final prosthesis was planned with full mouth prosthetic rehabilitation and a canine-protected occlusion using the PMS philosophy. This philosophy is used to obtain anterior guidance based on aesthetics and phonetics, followed by harmonious posterior disocclusion on protrusion and lateral excursive movements.

Mounting of the master cast to the semi-adjustable articulator (Hanau Wide Vue)

The maxillary master cast was mounted to the upper member of the articulator with the help of a Hanau Springbow earpiece face-bow. To mount the mandibular cast to the lower member of the articulator, the provisional crowns were removed step by step, starting from the right posterior section of the upper and lower quadrants. The interocclusal record material [10] was placed between the prepared teeth on the right side, while the provisional restoration on the left posterior acted as a stop. Similarly, the provisional crowns were removed from the left side, ensuring that the provisional restorations on the right side and the anterior teeth were kept intact, which acted as an occlusal stop. The interocclusal check record [10] was placed between the prepared teeth on the left side. A similar technique was used for the anterior segment. Finally, the three-segment interocclusal check record was used as a guide to mount the mandibular cast. A provisional cement was used to cement the provisional crowns to the prepared teeth.

**Figure 8:**
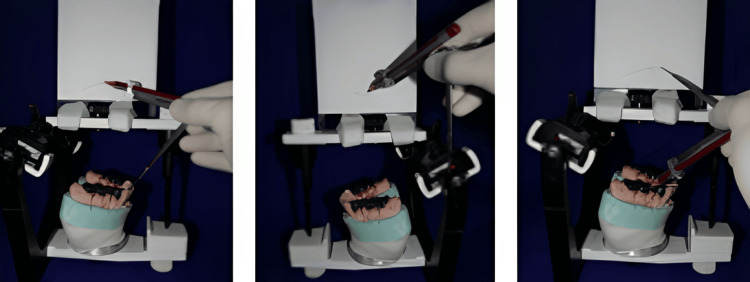
Broadrick’s occlusal plane analysis for anterior survey line, condylar survey line, and intersecting point (between anterior and posterior survey line).

After mounting the master cast, inlay pattern wax was used to fabricate the individual wax pattern copings following the cutback technique in the buccal aspect of the posterior teeth. Broadrick’s flag analysis was conducted for the posterior occlusal plane (Figure 8).

**Figure 9:**
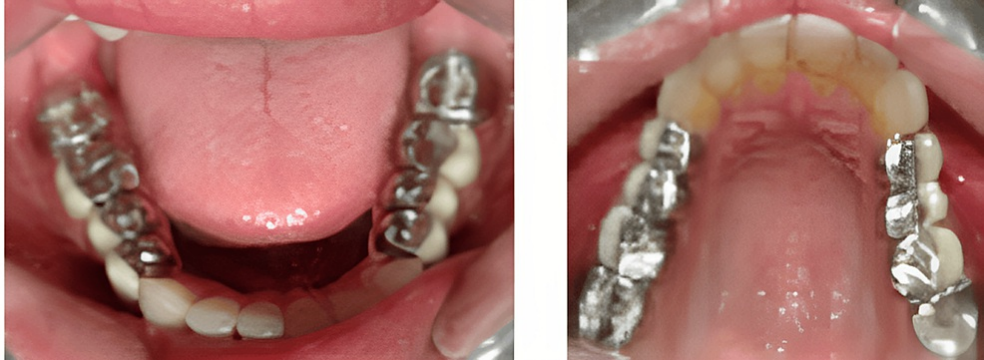
Permanent cementation of the mandibular porcelain-fused-to-metal (PFM) with ceramic facing with Broadrick’s flag analysis, followed by maxillary permanent cementation of PFM crowns.

The cutback technique for wax copings was used so the ceramic facing would be in the buccal aspect of the crown. The wax pattern was metal casted, finished, polished, and, finally, tried in the patient’s mouth to ensure a precise fit on the margins of the prepared teeth. The metal copings were refitted to the master cast, which was mounted on the semi-adjustable articulator, and the ceramic facing buildup was done. Ceramic facing in the buccal aspect prevents the clicking sound created by opposing ceramic teeth. The posterior occlusal morphological structure was made of metal, with porcelain only in the buccal aspect as a facing, to recontour the morphology of the posterior crowns (Figure 9).

**Figure 10:**
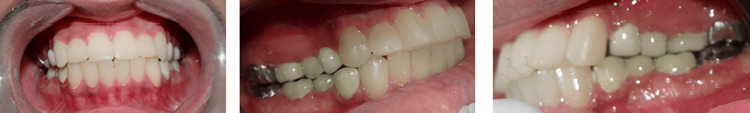
Frontal, right lateral, and left lateral views after cementation of the prosthesis in centric relation.

Occlusal morphology was reverified with Broadrick’s flag analysis for the posterior occlusal plane, which was made from the wax mockup of the diagnostic cast. Centric occlusion with maximum intercuspation was ensured (Figure 10).

**Figure 11:**
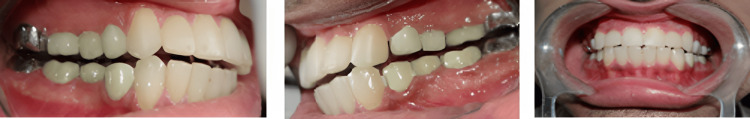
Posterior disocclusion (canine-guided occlusion) on lateral eccentric movements and protrusion.

It was also ensured that disocclusion of the left and right posterior teeth was achieved on left and right eccentric movements, with only canines coming into contact, which is canine-guided occlusion and posterior disocclusion on protrusion (Figure 11).

**Figure 12:**
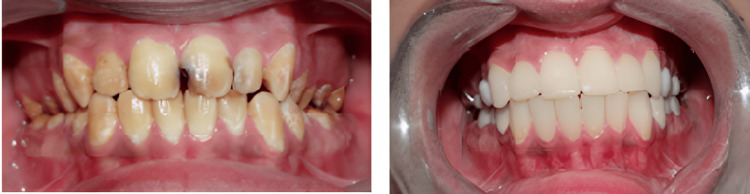
Preoperative and postoperative intraoral views of the patient.

Finally, in the fourth appointment, the PFM crowns were cemented to the prepared posterior teeth using type I glass ionomer cement. The occlusion was adjusted by removing interferences using the selective grinding technique. Finally, PFM crowns were glazed and permanently cemented to the prepared posterior teeth to ensure proper mastication, aesthetics, and phonetics (Figure 12).

**Figure 13:**
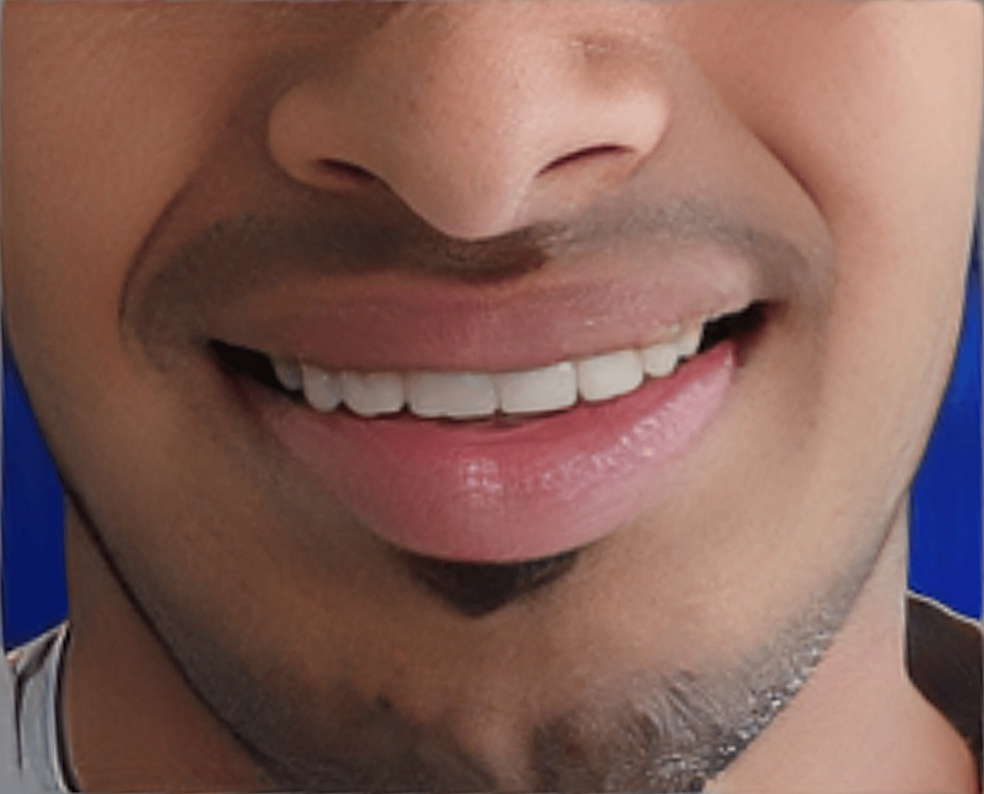
Healthy smile at six months of follow-up.

The patient was given instructions to maintain good oral hygiene, and regular follow-up was scheduled to ensure a good prognosis for the prosthesis. Follow-ups were done at two months, four months, and six months after cementation of the prosthesis. Oral hygiene was well maintained and the patient had a good periodontal condition of the prosthetically rehabilitated teeth with a healthy smile at six months of follow-up (Figure 13).

## Discussion

AGI is a rare disorder that manifests as generalized discoloration and wearing out of teeth, thereby leading to reduced VD and unaesthetic appearance with reduced chewing efficiency. Restoring the aesthetics, phonetics, and masticatory function of the patient through full mouth rehabilitation with anterior all-ceramic crowns and posterior PFM crowns with only buccal ceramic facing is an economic and aesthetic treatment modality for patients suffering from AGI [10]. Full mouth rehabilitation restores the patient’s confidence and self-esteem.

In this case, there was discoloration and wearing out of anterior and posterior teeth, leading to VD being reduced by 2 mm (collapsed bite), with an available free-way space of 6 mm. There was a loss of anterior guidance due to wear on the anterior teeth.

For full mouth rehabilitation of an AGI patient, the critical requirements are a healthy TMJ and harmonious anterior guidance, which should be in harmony with the posterior guidance as well. Any imbalance among these factors would hamper the treatment and have a negative impact on the stomatognathic system [11].

The diagnostic wax mockup must be done to visualize the aesthetic outcomes for the anterior teeth and any interferences in the centric and eccentric movements in the posterior teeth. The mockup also provides a clear picture of the necessary tooth preparation required for the treatment. In addition, provisional restorations can be made directly from the wax mockup using a putty index, which, in turn, saves chairside time.

The prosthetic rehabilitation of anterior teeth in PMS is based on anterior guidance for the best aesthetics, function, and comfort of the patient. Prosthetic rehabilitation of posterior teeth is based on posterior disocclusion and selection of standard occlusal pane, which would be in harmony with the incisal guidance.

Centric relation and incisal guidance play a crucial role in full mouth rehabilitation in the PMS philosophy technique. The incisal guidance is important in protecting the posterior teeth by disocclusion, in lateral eccentric and protrusive movements. Schluger et al. in 1971 proposed that there should be simultaneous interocclusal contact of posterior teeth in centric occlusion and posterior disocclusion in protrusive and lateral excursive movements and only canine coming in contact on the working side that is canine-guided occlusion [12].

Dadarwal et al. in 2022 explained the procedure involved in the prosthetic rehabilitation of an AGI patient, following Hobo’s twin-stage technique [13]. Dadarwal et al. in 2023 also elaborated on Hobo’s twin-stage technique of full mouth rehabilitation to rehabilitate a patient with generalized attrition of teeth [14]. This case report describes the procedure involved in full mouth rehabilitation of an AGI patient following the PMS philosophy.

A study by Thimmappa et al. in 2021 stated that no philosophies are universally applicable, although the most commonly accepted and used philosophy is PMS as it is based on a very flexible concept in which the incisal guidance is not governed by condylar guidance, but rather posterior occlusal plane is based on the anterior guidance for best esthetics, phonetics, function, and comfort of the patient [7].

This treatment option improves the patient’s quality of life and builds self-confidence. This treatment modality is an acceptable treatment for functional and aesthetic rehabilitation.

## Conclusions

Full mouth rehabilitation in a patient with AGI is a great challenge for the prosthodontist (operator). For a successful prosthetic rehabilitation, the operator needs to have a thorough knowledge of the concepts of occlusion in natural dentition (canine-guided or group function), the plane of occlusion, anteroposterior and mediolateral curves seen in natural dentition, centric and vertical jaw relation, hinge axes, and more. By following the PMS philosophy, treatment can be finished easily and comfortably, which is better for the patient. Our patient appreciated the clinical outcome of the procedure. The treatment was completed with enhanced aesthetics, phonetics, and masticatory function, which increased the patient’s confidence and self-esteem.
